# Flavour-active wine yeasts

**DOI:** 10.1007/s00253-012-4370-z

**Published:** 2012-09-01

**Authors:** Antonio G. Cordente, Christopher D. Curtin, Cristian Varela, Isak S. Pretorius

**Affiliations:** 1 The Australian Wine Research Institute, PO Box 197, Glen Osmond, Adelaide, SA 5064 Australia; 2 University of South Australia, GPO Box 2471, Adelaide, SA 5001 Australia

**Keywords:** Aroma, Flavour, Fermented beverages, Wine, Yeast

## Abstract

The flavour of fermented beverages such as beer, cider, saké and wine owe much to the primary fermentation yeast used in their production, *Saccharomyces cerevisiae*. Where once the role of yeast in fermented beverage flavour was thought to be limited to a small number of volatile esters and higher alcohols, the discovery that wine yeast release highly potent sulfur compounds from non-volatile precursors found in grapes has driven researchers to look more closely at how choice of yeast can influence wine style. This review explores recent progress towards understanding the range of ‘flavour phenotypes’ that wine yeast exhibit, and how this knowledge has been used to develop novel flavour-active yeasts. In addition, emerging opportunities to augment these phenotypes by engineering yeast to produce so-called grape varietal compounds, such as monoterpenoids, will be discussed.

## Introduction

While purchase of bottled wine is strongly influenced by extrinsic factors such as price (Mueller et al. 2010) and grape variety (King et al. 2012), the intrinsic flavour properties of a wine have a direct impact on how much it is ‘liked’ by consumers (Lattey et al. 2010). The ability to modulate wine style through changed winemaking practice is, therefore, an attractive target, that is dependent upon understanding flavour compound composition and how this influences sensory perception (Francis and Newton 2005). Nykanen (1986) reviewed progress in the field of wine and distillate flavour compound formation over a 25-year period (1960s–1980s), highlighting that while once it was thought alcoholic beverage flavours were composed of a small number of compounds, by 1985 more than 1,300 volatile compounds had been implicated. Many volatiles in wine are grape-derived, or form during processing and maturation — indeed the proportion of wine volatiles modulated by yeast was recently found to be relatively small (Robinson et al. 2011). Nonetheless, Nykanen (1986) contended that “the body of flavour is formed during fermentation by yeast”, and that “formation of the most dominant compounds occurring in beverages depend more on the yeast selected than the raw materials used in fermentation”. Ensuing research over the past 25 years has served to reinforce these observations.

The flavour compounds underlying the so-called 'yeast bouquet'; ethyl esters, acetate esters, fusel alcohols, carbonyls, and volatile fatty acids, are secondary metabolites synthesized by a wide range of microbial species. Depending upon winemaking practices, multiple yeast species from the grapes and winery equipment can be involved in alcoholic fermentation, and potentially contribute to wine flavour (Romano et al. 2003). Wine fermentation is a highly selective environment, however, and as ethanol concentrations rise, the species diversity of the ecosystem is diminished, giving way to predominance of the wine yeast, *Saccharomyces cerevisiae* (Heard and Fleet 1985). Consequently, most advancement in field over the past 25 years has been made in understanding formation of the core ‘yeast bouquet’ flavour compounds by *S. cerevisiae*, with production of esters (Saerens et al. 2010; Sumby et al. 2010) and fusel alcohols and acids (Hazelwood et al. 2008) recently reviewed.

Over the same 25-year period, *S. cerevisiae* emerged as the eukaryotic cell model system of choice, greatly enhancing the understanding of industrial yeast strains (Chambers and Pretorius 2010). Population genomics revealed a close relationship between man and yeast (Liti et al. 2009), and as useful industrial traits have been selected for over time, some consider that *S. cerevisiae* has been domesticated for brewing, baking, and winemaking (Legras et al. 2007; Verstrepen et al. 2006). Some industrially important phenotypes, such as ability to rapidly produce carbon dioxide (baking strains), ability to degrade maltose (brewing strains), ability to complete fermentation in high sugar grape musts (winemaking strains), are relatively straightforward to score and select for. Wine ‘flavour’ as a phenotype is much more ambiguous, but has nonetheless been a strong driver for wine yeast selection since the concept of single-yeast inoculation was introduced to the wine industry in 1890 (Pretorius 2000). Wines made through single-yeast inoculation differ in sensory properties to those made by spontaneous fermentations, an observation reinforced by differences in chemical composition (Varela et al. 2009). Hyma et al. (2011) recently found that domesticated *S. cerevisiae* strains made wines that were sensorially distinct from wines made by inoculation with single ‘wild’ *S. cerevisiae* strains, implying that the ‘flavour’ phenotype has indeed been a target for wine yeast domestication. Even amongst commercial, or domesticated, wine strains of *S. cerevisiae*, different wine flavour profiles generated solely through choice of yeast inoculum (including single, or multi-strain co-inoculation) can be detected by trained panels and wine professionals (King et al. 2008; Swiegers et al. 2009), and most importantly, by wine consumers (King et al. 2010).

**Fig. 1:**
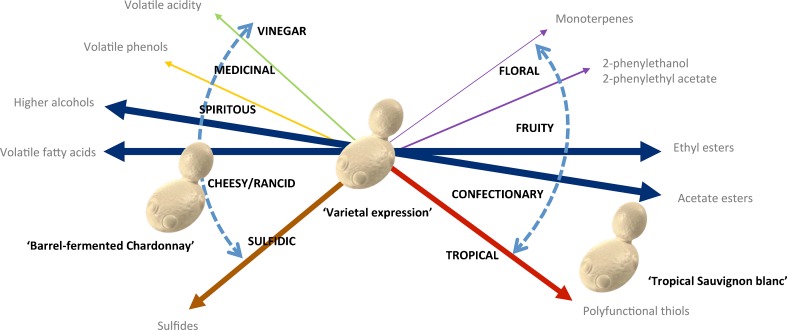
‘Flavour phenotypes’ that wine yeast have been selected for. Spectrum of flavour phenotypes that wine yeast exhibit (*bold, dashed arrows*), with flavour compound groups that drive them indicated by *solid arrows* weighted according to magnitude of impact. Examples of ‘flavour phenotypes’ that may be desirable for different winemaking objectives shown by positioning of yeast

What are the yeast ‘flavour phenotypes’ that have been selected for? In broad terms, wine yeast strains can be categorized on one dimension as 'fruity'–'floral', 'neutral', or 'cheesy'–‘rancid’–spirituous’, depending on their relative capacity to produce esters, higher alcohols, and volatile fatty acids (Fig. 1). Generally, there is a high level of correlation between individual compounds within these broader classes; however, there are exceptions. Wine strains of *Saccharomyces bayanus* produce relatively high concentrations of 2-phenylethanol and 2-phenylethyl acetate compared to other higher alcohols and acetate esters, which may enhance ‘rose’ and ‘floral’ characters (Masneuf-Pomarede et al. 2010). An additional dimension can be used to separate wine yeast according to their production of sulfur containing compounds, which are associated with ‘tropical’ or ‘sulfidic’ flavours in wine (Fig. 1). Further 'flavour-fault' phenotypes include 'medicinal' phenolic off-flavour, and excessive production of volatile acidity —imparting a flavour associated with vinegar. Finally, some floral nuances can be imparted by yeast able to release glycosidically bound monoterpenes (Ubeda and Briones 2000; Ugliano et al. 2006), or, as a result of mutations in the ergosterol pathway, able to de novo synthesize these 'varietal' compounds at low levels (Chambon et al. 1990, 1991).

Some of the genetic and environmental factors that affect these phenotypes are well understood — the *PAD1* gene from *S. cerevisiae* was designated Pof through genetic studies that linked it to the phenolic off-flavour phenotype encountered in brewing (Goodey and Tubb 1982; Meaden and Taylor 1991), and Pof-wine yeast are available today. On the other hand, prior to 2005 there was no knowledge of *S. cerevisiae* genes involved in formation of polyfunctional thiols (Howell et al. 2005). Expanding the knowledge of enzymes involved in flavour compound formation, and the genetic networks that regulate them, is crucial to advancing the capability to develop flavour-active yeasts with the best mix of flavour phenotypes.

This review will explore recent advances in our understanding of yeast influence on formation of flavour compounds, then focus on the emerging opportunity to engineer wine yeast to enhance formation of so-called grape 'varietal' flavour compounds, such as the monoterpenoids and high-impact sulfur-containing polyfunctional thiols.

## Recent advances in knowledge of flavour compound formation by yeast

### Ethyl esters and acetate esters

There are two classes of flavour-active esters in fermented beverages. First, the acetate esters, where the acyl group is derived from acetate (in the form of acetyl-CoA), and the alcohol group is ethanol or a complex alcohol derived from amino acid metabolism. The most significant acetate esters are ethyl acetate (‘fruity’, ‘solvent-like’ aromas), isoamyl acetate (‘banana’ aroma), and 2-phenylethyl acetate (‘honey’, ‘roses’, ‘flowery’ aromas). The second group comprises the medium-chain fatty acid (MCFA) ethyl esters, where the alcohol group is ethanol, and the acyl group is derived from activated medium-chain fatty acids. Examples are ethyl hexanoate (‘apple-like’ aroma), and ethyl octanoate (‘apple’ aroma).

The rate of ester formation during fermentation is dependent on two primary factors: (1) the concentration of the co-substrates, the acyl-CoA and the alcohol; and (2) the activity of enzymes involved in their synthesis and hydrolysis (acyltransferases and esterases) (Saerens et al. 2006, 2008; Verstrepen et al. 2003). To date, five distinct proteins — Atf1p, Atf2p, Eht1p, Eeb1p and Iah1p — have been identified and characterized in *S. cerevisiae* as having ester synthesis or hydrolysis activity, with the alcohol acetyltransferase Atf1p having the greatest activity and being the most studied (Lilly et al. 2000, 2006a; Saerens et al. 2010; reviewed by Sumby et al. 2010; Verstrepen et al. 2003).

Overexpression of *ATF1* during wine fermentation results in a significant increase (between 10- and 200-fold) in acetate ester production (Lilly et al. 2000, 2006a; Verstrepen et al. 2003), whereas *ATF2* appears to play a minor role in ester formation (Lilly et al. 2006a; Verstrepen et al. 2003). Excessively high production of ethyl acetate by yeast overexpressing *ATF1* did not improve the fermentation bouquet and aroma of the young wines, however, it was observed that hydrolysis during bottle aging caused a significant decrease in the levels of acetate esters, particularly ethyl acetate (Lilly et al. 2000). Therefore, higher initial levels of esters could lead to wines with a more fruity character (Lilly et al. 2000). On the other hand, the deletion of both *ATF1* and *ATF2* completely abolishes the formation of isoamyl acetate (Verstrepen et al. 2003). However, the double deletion strain still produced about 50 % as much ethyl acetate the wild-type strain, suggestive of the existence of unknown acetate ester synthases in the yeast genome.

Acetate ester formation by yeast is balanced by the *IAH1*-encoded esterase. Diploid brewer’s yeast strains deficient in *IAH1* accumulate much higher amounts of isoamyl acetate than do the parent strains (Fukuda et al. 1998). Conversely, overexpression of *IAH1* results in a significant decrease in the concentration of many esters, including isoamyl acetate, hexyl acetate, ethyl acetate, and 2-phenylethyl acetate, compared to control strains (Lilly et al. 2006a). *IAH1* crucially regulates the accumulation of isoamyl acetate and other esters during fermentation and thus determines the flavour quality of wine (Lilly et al. 2006a). Recently, Iah1p has been crystallized (Ma et al. 2011). The hydrolytic activity of Iah1p was shown to be maximal for acetate esters, and was lower with hexanoate esters. Interestingly, a C-terminally truncated version of Iah1p was able to hydrolyze decanoate esters.

A further, largely unexplored, level at which acetate ester formation is modulated is the availability of acetyl-CoA. Increased levels of both CoA and acetyl-CoA were accompanied by a 6-fold increase in production of isoamyl acetate, for an *Escherichia coli* strain expressing the yeast *ATF2* gene and overexpressing its own pantothenate kinase (*panK*) gene — which regulates CoA biosynthesis (Vadali et al. 2004). A decrease in formation of isoamyl acetate and ethyl acetate by *S. cerevisiae* was noted when carnitine acetyltransferase, an enzyme that regulates the transfer of activated acetyl groups to the mitochondria and regulates acetyl-CoA/CoA pools within the cells, was overexpressed (Cordente et al. 2007).

*EHT1* and *EEB1* encode proteins with MCFA ethyl ester synthase and esterase activities. Specifically, *EEB1* encodes an ethanol acyltransferase responsible for the synthesis of the majority of MCFA ethyl esters during fermentation (Saerens et al. 2006), while *EHT1* encodes for an ethanol hexanoyl transferase, which plays a minor role in MCFA ethyl ester biosynthesis. Both *EHT1* and *EEB1* also possess short-chain esterase activity (Saerens et al. 2006). Another protein encoded by *YMR210w*, and similar to both Eeb1p and Eht1p, has been identified as a putative acyltransferase (Saerens et al. 2006). Whereas expression levels seem to be the limiting factor for *ATF1* gene regulation and acetate ester production, this does not seem to be the case for *EEB1* and *EHT1* — overexpression of these genes in an industrial strain had a minor effect on MCFA ethyl ester content, presumably due to their competing synthesis and hydrolysis activities (Lilly et al. 2006a; Saerens et al. 2006). Precursor availability appears to be the limiting factor in ethyl ester biosynthesis (Saerens et al. 2008) since the addition of hexanoic or octanoic acid to the fermentation medium causes a strong increase in the formation of the corresponding ethyl ester (Saerens et al. 2006). In this regard, octanoic (but not hexanoic) acid induces expression of *EEB1* and *EHT1* (Saerens et al. 2008). Recently, a transcriptomic analysis has revealed that *EEB1* was the most strongly induced gene (8.4-fold) after addition of decanoic acid (Legras et al. 2010), which suggests that ethyl ester synthesis plays a complementary role in the detoxification of MCFA. A slight (35 %) but significant induction of *YMR210w* was also reported in these conditions.

The possible role of *YMR210w* in ethyl ester synthesis remains unclear. While overexpression of this open reading frame (ORF) does not significantly affect the concentration of ethyl esters at the end of fermentation (Rossouw et al. 2008; Saerens et al. 2006), its native expression levels correlate positively with ethyl acetate, ethyl octanoate and isoamyl acetate (Rossouw et al. 2008). Furthermore, while deletion of *YMR210w* does not affect the production of MCFA ethyl esters, deletion of this gene in an *Δeeb1* or *Δeeb1Δeht1* background further decreases formation of both ethyl octanoate and ethyl decanoate.

### Higher alcohols and volatile fatty acids

Alcoholic fermentation is also accompanied by the formation of aliphatic and aromatic alcohols known as higher alcohols or fusel alcohols. While fusel alcohols at high concentrations impart off-flavours, low concentrations of these compounds and their esters make a crucial contribution to the flavour and aroma of wine (Lambrechts and Pretorius 2000; Nykanen et al. 1977). In particular, 2-phenylethanol is considered to be one of the most important aromatic alcohols contributing to wine flavour. The higher alcohols are predominantly formed by yeast during fermentation from α-keto acids, involving degradation of an amino acid via the so-called Ehrlich pathway (Ehrlich 1904; reviewed by Hazelwood et al. 2008; Styger et al. 2011b), but can also be synthesised from glucose via pyruvate (Chen 1978; Dickinson et al. 1997; Eden et al. 2001). The Ehrlich pathway involves three steps: (1) an initial transamination that results in the formation of an α-keto acid; (2) decarboxylation of the α-keto acid to form a ‘fusel aldehyde’; and (3) its reduction to generate the ‘fusel alcohol’.

Four *S. cerevisiae* genes have been implicated in the transamination step of the Ehrlich pathway: the mitochondrial and cytosolic branched-chain amino acid (BCAA) aminotransferases (*BAT1* and *BAT2*, respectively) and the aromatic amino acid aminotransferases I and II (*ARO8* and *ARO9*, respectively) (Eden et al. 1996; Iraqui et al. 1998; Kispal et al. 1996). Researchers have looked at the effect of modulating yeast BCAA activity on the production of higher alcohols (Eden et al. 2001; Lilly et al. 2006b). In wines and distillates, the overexpression of *BAT1* increased the concentration of isoamyl alcohol, its acetate ester, as well as isobutanol; while overexpression of *BAT2* resulted in a substantial increase in the formation of isobutanol and isobutyric acid (Lilly et al. 2006b). Sensory analysis confirmed that the overexpression of *BAT1* and *BAT2* had an impact on aroma profiles of wines and distillates (Lilly et al. 2006b). The perturbation of the *BAT* genes not only affects the concentrations of metabolites directly linked to these genes, but also other aroma metabolites not directly related to higher alcohols, highlighting the complexities of the interconnections within such complex metabolic networks (Lilly et al. 2006b; Styger et al. 2011a). *BAT2* seems to have a more prominent role than *BAT1* in the Ehrlich pathway. In support of this hypothesis, it has been recently shown that *BAT2* function is determinant for BCAA catabolism, while *BAT1* is involved in the biosynthesis of these amino acids (Colon et al. 2011).

To date, five proteins have been implicated in α-keto decarboxylation: the pyruvate decarboxylases Pdc1p, Pdcp5*,* and Pdc6p; the phenylpyruvate decarboxylase Aro10p; and the probable carboxylase Thi3p (Styger et al. 2011a), which plays a role as a regulatory protein of the enzymes involved in thiamine biosynthesis (Mojzita and Hohmann 2006; Nosaka et al. 2005).

The final step of the Ehrlich pathway involves either the reduction or oxidation of the fusel aldehydes to form fusel alcohols or fusel acids, respectively. Formation of the fusel alcohols can be catalyzed by several oxidoreductases: the alcohol dehydrogenases (Adh1p to Adh7p) (Dickinson et al. 2003; Kondo et al. 2012; Larroy et al. 2002), the formaldehyde dehydrogenase Sfa1p (Dickinson et al. 2003), the 3-methylbutanal reductase Gre2p (Hauser et al. 2007), and the NADPH-dependent aldo-keto reductase Ypr1p (Ford and Ellis 2002), and at least one of the putative aryl-alcohol dehydrogenases (*AAD6*) (Styger et al. 2011a). The balance between oxidation and reduction of the fusel aldehydes depends on the global redox status of the yeast cell. In glucose-grown batch cultures of *S. cerevisiae*, where growth is predominantly fermentative, the formation of fusel alcohols is favoured over that of the acids (Dickinson et al. 1997, 2003), while the opposite is true in aerobic-limited chemostat cultures grown in the presence of various amino acids (Vuralhan et al. 2003).

Recently, Styger et al. (2011a) conducted a targeted screen of genes encoding dehydrogenase, decarboxylase and reductase enzymes potentially involved in flavour compound formation via the Ehrlich pathway. The ten genes with greatest impact on higher alcohol formation were further characterized, including some not previously linked with this pathway: two highly promiscuous carboxylases (*PAD1* and *SPE1*) and two dehydrogenases (*OYE2* and *HOM2*)*.* Discovery of novel flavour-active genes such as these provide excellent targets for biotechnological improvement of aroma production by industrial strains of *S. cerevisiae*.

### Strategies to modify ester, higher alcohol and volatile fatty acid flavour profiles of yeast

Recently, several strategies for genetic engineering of *S. cerevisiae* to increase productivity of isobutanol from glucose through the endogenous Ehrlich pathway have been reported (Chen et al. 2011; Kondo et al. 2012). Kondo enhanced the Ehrlich pathway activity by overexpressing several combinations of alcohol dehydrogenases and keto-acid decarboxylases. Overexpression of the medium-chain alcohol dehydrogenases *ADH6* and *ADH7* displayed higher isobutanol productivities, as did the overexpression of the probable decarboxylase *THI3*. The production of isobutanol was further improved by altering carbon flux towards valine biosynthesis and deleting the pyruvate decarboxylase *PDC1.* Chen et al. (2011) used a different strategy based upon the overexpression of several genes involved in valine biosynthesis, along with *BAT2*, achieving similar results.

Another strategy to increase the formation of higher alcohols, specifically 2-phenylethanol (‘flowery’, ‘rose’ aroma), is the expression in yeast of flower and fruit enzymes involved in the production of this aromatic volatile. Farhi et al. (2010) demonstrated that yeast can be harnessed in the field of floral volatiles by expressing the rose phenylacetaldehyde synthase, which was shown to complement the deletion of the native phenylpyruvate decarboxylase *ARO10*, and to enhance the production of both the alcohol and phenylacetaldehyde compared to the wild-type strain.

To date, there has been almost no application of genetically modified (GM) technology in commercial winemaking (Chambers and Pretorius 2010; Pretorius et al. 2012); therefore, non-GM strategies to develop flavour-active yeast are required. The isolation of yeast mutants, induced or spontaneous, that are resistant to different drugs and amino acids analogues, has proven an effective strategy for modulation of ester production by yeast (Fukuda et al. 1990a, b; Hirooka et al. 2005; Ichikawa et al. 1991). It has been reported that mutant saké yeast resistant to cerulenin, an inhibitor of fatty acid synthesis, overproduced ethyl hexanoate, one of the most important components of saké flavour (Ichikawa et al. 1991). Cerulenin resistance is conferred by a particular dominant mutation in the fatty synthase (*FAS2*) gene (Fas2p^G1250S^) (Inokoshi et al. 1994). A self-cloning saké strain bearing this mutation, and no extraneous DNA sequences, has become the first GM microorganism to be approved for use in Japan (Aritomi et al. 2004).

Saké yeast mutants resistant to the l-leucine analog 5,5′,5″-trifluoro-dl-leucine (TFL) (Ashida et al. 1987; Oba et al. 2005) show higher levels of isoamyl acetate production. Resistance to TFL has been linked to mutations in *LEU4* gene (Casalone et al. 1997; Oba et al. 2005), which releases leucine feedback inhibition and causes hyper-production of isoamyl alcohol, and thus, an accumulation of the corresponding acetate ester. Saké yeast resistant to *o*- and *p*-fluoro-dl-phenylalanine produce higher levels of 2-phenylethanol and 2-phenylethyl acetate (Fukuda et al. 1990a, b). Hirooka et al. (2005) isolated a spontaneous saké mutant resistant to 1-farnesylpyridinium, an analog of the isoprenoid farnesol (Hirooka et al. 2005), with improved production of isoamyl acetate. This mutant has an increased alcohol acetyltransferase activity, and it is currently used for industrial saké brewing (Hirooka et al. 2010).

While much research in this area has been devoted to industrial yeast for saké brewing, cerulenin and TFL resistant *S. cerevisiae* yeast strains used in the production of cachaça, the Brazilian sugarcane spirit, have been isolated that produce higher levels of both isoamyl acetate and ethyl hexanoate (de Souza et al. 2012; Vicente et al. 2006). Similar strategies have not, to date, been applied in development of flavour-active wine yeast. Ichikawa et al. (1991) noted that ethyl hexanoate overproduction by their *FAS2* mutant saké yeast was accompanied by an increase in formation of hexanoic acid — while this may not be detrimental to saké quality, the net effect of similar mutations in wine yeast on flavour profile and balance awaits evaluation.

### Monoterpenoids

Terpenoids (isoprenoids) comprise a large and diverse family of naturally occurring compounds, which are involved in the fragrance and aroma of flowers and fruits, plant defense and primary plant metabolism. All terpenoids are synthesized from the universal five carbon precursors, isopentenyl diphosphate (IPP) and dimethylallyl diphosphate (DMAPP). The significance of volatile C10 monoterpenes to the flavour and varietal character of some cultivars of *Vitis vinifera* is well reviewed (Mateo and Jimenez 2000; Strauss et al. 1986; Versini et al. 1999).

Monoterpenes are present as free as well as glycosylated flavourless conjugates amongst the secondary metabolites of certain grape varieties of *V. vinifera*. Hence, when these compounds are detected in wine they are considered to originate from grape and not from fermentation. In general, more bound glycosides are found than free terpenoids, and the ratios of bound to free terpenoids can also vary amongst different grape cultivars (Williams et al. 1984). Both bound and free terpenoids can be modified to various degrees during alcoholic and malolactic fermentation (Swiegers et al. 2005). During winemaking, bound terpenoids can be released by glycosidase enzymes produced by grapes, yeast and bacteria, increasing the volatile terpenoid composition of wines and enhancing wine aroma and flavour (van Rensburg and Pretorius 2000). Enzymatic hydrolysis of glycosides occurs in two steps: first, depending on the diglycoside conjugate, either an α-l-arabinofuranosidase, an α-l-rhamnosidase or a β-d-apiosidase release the corresponding monoterpenyl glucosides. Second, monoterpenyl glucosides are then hydrolysed by the action of a β-glucosidase releasing the monoterpene alcohol (Flipphi et al. 1993; LeClinche et al. 1997; Ramachandran et al. 2012; Zietsman et al. 2011). β-Glucosidases do not have endoglucanase activity and therefore can only act on monoterpenyl glucosides (Gunata et al. 1988).

Several *S. cerevisiae* strains have been shown to secrete enzymes characterised principally by β-glucosidase activity (Fernandez et al. 1999; Ubeda and Briones 2000; Ugliano et al. 2006). However, their activity towards monoterpenyl glycosides is very low (Hernandez et al. 2003). A well-recognised strategy to improve the hydrolysis of glycosylated bound conjugates is the addition of exogenous enzyme preparations from other microorganisms during or after fermentation (Armada et al. 2010; Genoves et al. 2003; van Rensburg and Pretorius 2000; Vasserot et al. 1993). Commercial preparations contain a mix of pectinases, glucanases and xylanases obtained principally from *Aspergillus* spp. Addition of exogenous enzyme preparations can increase production costs; moreover, the lack of specificity of these enzymes might induce secondary reactions detrimental to wine flavour (Riou et al. 1998).

Another strategy to enhance formation of monoterpenes during winemaking is to engineer *S. cerevisiae* wine yeast by introducing enzymes able to hydrolyse glycosylated precursors (Manzanares et al. 2003; Pretorius and Bauer 2002; Schuller and Casal 2005). There are several reports in literature assessing the effect of the exogenous expression of these enzymes on the chemical composition and aroma profile of wines fermented with engineered strains. Expression of the β-(1,4)-endoglucanase encoded by the *egl1* gene from *Trichoderma longibrachiatum* changed volatile composition and enhanced perception of fruity aroma (Perez-Gonzalez et al. 1993). Engineered strains expressing the *Aspergillus nidulans xlnA* gene encoding for a β-(1,4)-endoxylanase showed significative higher concentrations of several esters, higher alcohols and terpenes, particularly, ethyl acetate, 3-methyl butanol, 2-phenylethanol and linalool in Chenin Blanc wines (Ganga et al. 1999). Manzanares et al. (2003) engineered two wine strains: one expressed the α-rhamnosidase gene (*rhaA*) from *Aspergillus aculeatus* and the second expressed the β-glucosidase gene from *Candida molischiana*. Wines co-fermented with both strains showed an increase in the concentration of linalool, α-terpeniol, nerol and geraniol in Muscat wine. Expression of β-glucosidases from *Saccharomycopsis fibuligera* showed not only higher levels of terpenols but also increased concentrations of esters (van Rensburg et al. 2005). Gil et al. (2005) overexpressed the *S. cerevisiae* exoglucanase encoded by the *EXG1* gene. Wines fermented with engineered strains exhibited the higher concentrations of volatile compounds, including several alcohols and terpenols. Co-expression of the *xyn2* gene from *Trichoderma reesei* which encodes a xylanase and the *end1* gene from *Butyrivibrio fibriosolvens* encoding an endo-β-(1,4)-glucanase showed significant improvement in the aromatic profile of wines fermented by engineered strains (Louw et al. 2006). Zietsman et al. (2011) constructed a wine yeast co-expressing an α-l-arabinofuranosidase from *Aspergillus awamori* and a β-glucosidase from *S. fibuligera*. Gewürztraminer wine fermented with the engineered strain showed significative higher concentrations of linalool, citronellol, nerol and α-terpineol and lower concentration of geraniol after fermentation, and resulted in wines exhibiting higher floral and fruity characters than non-engineered wine.

Although genetic engineering approaches can considerably change the volatile composition and enhance the varietal aroma profile of wine they are not used in the commercial production of wine. Therefore, attention has been focussed on the characterisation and development of non-genetically modified wine strains able to increase the release of monoterpenes (Fernandez-Gonzalez et al. 2003; Gamero et al. 2011; Hernandez-Orte et al. 2008).

All the strategies described above, however, are less useful for musts derived from non-aromatic grape varieties having low contents of free and bound monoterpenes. An alternative would be to engineer wine yeast for the *de novo* biosynthesis of monoterpenes through the existing mevalonate pathway, which results in the formation of IPP and DMAPP.

Unlike plants, *S. cerevisiae* cannot produce monoterpenes efficiently, and only a few natural *S. cerevisiae* strains have been shown to produce small amounts of monoterpenes (Carrau et al. 2005; Zea et al. 1995). This is because *S. cerevisiae* lacks enzymes with monoterpene synthase activity (MTS), which catalyze the conversion of the universal precursor, geranyl diphosphate (GPP) to monoterpenes. In addition, yeast do not carry a specific GPP synthase, and this metabolite only occurs as an intermediate of farnesyl diphosphate (FPP) synthesis, which is the precursor of several classes of essential metabolites such as ergosterol, ubiquinone, dolichols, or heme A (Grabinska and Palamarczyk 2002). In yeast, GPP and FPP synthase activities are shared by one enzyme: farnesyl diphosphate synthase (FPPS). FPPS catalyzes two sequential condensation reactions of the IPP with its isomer DMAPP into GPP, and then GPP with another IPP molecule into FPP. It was thought that tight binding of GPP to the FPPS catalytic site might lead to minimal release of GPP for biosynthesis of monoterpenoids. However, it has been established that *S. cerevisiae* has enough free GPP to be used by exogenous MTS to produce monoterpenes under laboratory and vinification conditions (Herrero et al. 2008; Oswald et al. 2007).

The *ERG20*-encoded FPPS enzyme is essential for *S. cerevisiae*. Yeast mutants secreting the monoterpene alcohols linalool and geraniol have been characterized previously (Chambon et al. 1990, 1991), which carry a specific mutation in *ERG20* (Erg20p^K197E^) (Blanchard and Karst 1993). This mutation leads to an increase of the available GPP for monoterpene synthesis (Blanchard and Karst 1993; Fischer et al. 2011). Therefore, interrupting the sterol pathway by mutation in *ERG20* can alter monoterpene content.

In recent years, many genes have been characterized that encode plant MTS, for example; the linalool synthase gene from *Clarkia breweri* (Dudareva et al. 1996), the geraniol synthase from *Ocimum basilicum* (Iijima et al. 2004), and α-terpineol synthase from *V. vinifera* (Martin and Bohlmann 2004). Since all monoterpenes are produced from the ubiquitous C10 intermediate GPP, it is possible to engineer yeast for the de novo production of specific monoterpene(s). The introduction of MTS in yeast leads to a redirection of the flux of the isoprenoid precursors DMAPP and IPP towards GPP, competing with FPP formation, which is required to produce sterols (Herrero et al. 2008). Yeast has been harnessed in several recent studies to act as a cell factory for production of different terpenes (Farhi et al. 2011; Fischer et al. 2011; Herrero et al. 2008; Oswald et al. 2007; Rico et al. 2010; Tokuhiro et al. 2009), as reviewed by Siddiqui et al. (2012).

An emerging opportunity to engineer wine aroma has arisen through recent work characterizing novel MTS encoding genes from *V. vinifera* (Martin et al. 2010). Recent analysis of the grapevine genome allowed the prediction of 69 putatively functional terpene synthase (VvTPS) encoding genes, which represent five of the seven plant TPS subfamilies. In addition, 39 of these VvTPS enzymes were functionally characterized, the largest number of TPS characterized for any species, and found to produce different profiles of terpenoids. As other grapevine genome sequences become available, the number of available MTS genes will grow, further expanding the potential for engineering of *S. cerevisiae* to produce terpene profiles to achieve desired sensory profiles in finished wines.

### Volatile sulfur compounds

The propensity of *S. cerevisiae* yeast to produce negative volatile sulfur compounds (VSCs), particularly ‘rotten-egg’-aroma imparting hydrogen sulfide (H_2_S), has been well studied (reviewed by Swiegers and Pretorius 2007). VSCs in wine can be considered a ‘double-edged sword’, as some sulfur-containing flavour compounds contribute positively to wine (Swiegers and Pretorius 2005). Prominent examples include furfurylthiol (‘roast coffee’ aroma) (Tominaga et al. 2000); and the ‘fruity’ polyfunctional thiols 3-mercaptohexan-1-ol (3MH), 4-mercapto-4-methyl-pentan-2-one (4MMP), and 3-mercaptohexyl acetate (3MHA), that impart ‘passionfruit’, ‘grapefruit’, ‘gooseberry’, ‘guava’, and ‘box hedge’ aromas (Dubourdieu et al. 2006; Swiegers et al. 2006; Swiegers and Pretorius 2005). Other important VSCs found in wine include methanethiol (‘cooked cabbage’ aroma); dimethylsulfide, dimethyldisulfide, and dimethyltrisulfide (‘cabbage’, ‘cauliflower’, and ‘garlic’ aromas); and methylthioesters (‘cooked cauliflower’, ‘cheesy’ and ‘chives’ aromas).

The production of H_2_S is a significant problem for the global wine industry since it imparts an undesirable ‘sulfurous’, ‘rotten egg’-like off-flavour (Rauhut 1993), even at low concentrations (1 μg/l) (Siebert et al. 2009). The production of H_2_S during wine fermentation is a frequently encountered problem in winemaking, and, if it is not treated, the resulting wine will be tainted leading to a loss in quality and the possibility of being rejected by consumers. It is well established that *S. cerevisiae* is responsible for H_2_S off-flavour in wine and that the production is strain dependent (Acree et al. 1972; Giudici and Kunkee 1994; Kumar et al. 2010; Mendes-Ferreira et al. 2002; Nowak et al. 2004), even though not all wine yeast produce H_2_S — about 1 % of naturally occurring wine strains are unable to produce this off-flavour (Zambonelli et al. 1984). Other factors affecting the production of H_2_S include environmental and nutritional factors such as the availability of sulfur compounds (sulfur dioxide, organic sulfur compounds, and elemental sulfur in the vineyard for plant protection); nitrogen limitation, and vitamin deficiency (Giudici and Kunkee 1994; Rauhut 1993; Rauhut and Kurbel 1994; Spiropoulos et al. 2000; Ugliano et al. 2009; Wang et al. 2003; Winter et al. 2011a). H_2_S can be formed metabolically by wine yeast from inorganic sulfur compounds, sulfate, and sulfite, or organic compounds, cysteine, and glutathione (Henschke and Jiranek 1993; Rauhut 1993; Spiropoulos et al. 2000). The majority of H_2_S produced during winemaking occurs as a result of the biosynthesis of the sulfur containing amino acids methionine and cysteine, which occur in low concentrations in grape juice, through the *sulfate reduction sequence* (SRS). These amino acids are essential for the growth of *S. cerevisiae,* and if they are not present, or depleted, in the growth medium, then sulfur must be assimilated from inorganic sources (Henschke and Jiranek 1993). The most common sulfur source in *S. cerevisiae* is extracellular sulfate, which naturally exists in high amounts in grape juice (Vos and Gray 1979).

In the first step of the SRS pathway, sulfate is transported into the cell by two specific permeases before a two-step activation with the aid of two molecules of ATP. The first reduction step produces sulfite, which is, in turn, reduced by sulfite reductase to sulfide. At this point, the sulfide produced is combined with a nitrogenous precursor, *O*-acetyl serine or *O*-acetyl homoserine, to ultimately form cysteine and methionine. If there is a deficiency of assimilable nitrogen in the grape must, *O*-acetyl serine or *O*-acetyl homoserine becomes limiting, and sulfide builds up and is converted to the volatile gas H_2_S, which then diffuses from the yeast cell into the wine (Giudici and Kunkee 1994; Henschke and Jiranek 1993).

Several genetic engineering strategies have been used for limiting H_2_S production, which generally consisted in the overexpression or inactivation of some of the genes involved in the SRS pathway. Constitutive expression of the *MET25* gene (alias *MET17*), which encodes a bifunctional *O*-acetylserine/*O*-acetylhomoserine sulfhydrylase, lowered H_2_S by 2-fold in a brewing yeast (Omura et al. 1995). In a similar study, the overexpression of the same gene in a strain of *S. cerevisiae* greatly reduced H_2_S formation in a wine ferment, but this was not the case for another strain (Spiropoulos and Bisson 2000). Overexpression of the *CYS4* gene, encoding cystathionine β-synthetase, was also shown to reduce H_2_S production (Linderholm et al. 2006; Tezuka et al. 1992). Altering sulfite reductase activity has been considered a better approach for limiting H_2_S formation in yeast, since reducing the production of sulfide is a better approach than trying to consume it in a later metabolic step. The yeast NADPH-dependent sulfite reductase is a heterotetramer protein, consisting of two α- and two β-subunits (α_2_β_2_). The α-subunit is encoded by the *MET10* gene, whereas the β-subunit is encoded by the *MET5* gene. The inactivation of *MET10* in a brewer’s yeast resulted in increased sulfite accumulation during beer production and increased flavour stability, and no sign of H_2_S production (Hansen and Kielland-Brandt 1996). In wine yeast, there have been some efforts to develop commercial yeast with impaired hydrogen sulfide production (Cordente et al. 2009; Linderholm et al. 2010), in which the partial inactivation of either of the two catalytic subunits of the sulfite reductase enzyme led to the desired phenotype. Recently, the *MET10* G176A allele, present in one of the low-H_2_S strains described by Cordente et al. (2009), was found to have a strong dominant effect, which allowed the use of this strain in the breeding of new interspecific hybrids with a low-H_2_S production phenotype and other desired industrial traits (Bizaj et al. 2012).

The polyfunctional thiols 4MMP, 3MH and 3MHA are extremely potent having perception thresholds in the parts per trillion range (Dubourdieu et al. 2006; Tominaga et al. 1998a, b). These compounds are of particular importance for the varietal character of Sauvignon Blanc wines (reviewed by Coetzee and du Toit 2012), and are found to be highly desired in some styles of Sauvignon Blanc by consumers (King et al. 2012). It has been shown that 4MMP and 3MH exist in grapes in their non-volatile precursor form, conjugated to cysteine or glutathione (Fedrizzi et al. 2009; Peyrot Des Gachons et al. 2002; Roland et al. 2010a; Tominaga et al. 1998a). The wine yeast take up these precursors and cleave them to release the corresponding free thiol during fermentation (Darriet et al. 1995; Grant-Preece et al. 2010; Winter et al. 2011b), although only a small fraction of available precursors are converted to the respective polyfunctional thiols (Dubourdieu et al. 2006; Subileau et al. 2008b; Winter et al. 2011b). No cysteine or glutathione precursor of 3MHA has been identified, and this compound is formed during fermentation and through esterification of 3MH by the alcohol acetyltransferase *ATF1*. The overexpression of *ATF1* in both commercial and laboratory strains results in a significant increase in the amount of 3MHA formed, while the overexpression of the esterase *IAH1* had the opposite effect (Swiegers et al. 2006).

Yeast strains vary in their abilities to release polyfunctional thiols, and therefore selection of yeast strain is highly important to modulate their concentration in wine (Dubourdieu et al. 2006; Howell et al. 2005; Swiegers et al. 2006, 2009). Polyfunctional thiol production also depends on other factors, such as fermentation temperature (Masneuf-Pomarede et al. 2006; Swiegers et al. 2006), addition of nutrients to active dry yeast rehydration media (Winter et al. 2011a), pre-fermentation operations such as skin contact (Peyrot Des Gachons et al. 2002), as well as oxygen, phenol, and sulfur dioxide content (Blanchard et al. 2004).

The genetic determinants for release of 3MH and 4MMP from their cysteinylated precursors have been studied in a targeted manner over recent years (Holt et al. 2011; Howell et al. 2005; Roncoroni et al. 2011; Subileau et al. 2008a; Thibon et al. 2008). Uptake of the precursors is assumed to be mediated by amino acid transporters on the plasma membrane. However, the deletion of the general amino acid transporter, *GAP1,* has a limited effect on 3MH release from the cysteine precursor Cys-3MH in synthetic media (Subileau et al. 2008a), which indicates that other transporters might be involved in its uptake during fermentation. Once inside the cell, the cysteinylated precursor is cleaved by a yeast enzyme with carbon–sulfur β-lyase activity (Swiegers et al. 2007; Tominaga et al. 1995). A gene encoding a yeast β-lyase enzyme, *IRC7*, was found to be the key determinant of 4MMP release (Roncoroni et al. 2011; Thibon et al. 2008), while also contributing to the release of 3MH. Interestingly, most strains of *S. cerevisiae* (Liti et al. 2009; Roncoroni et al. 2011), have a deletion in the C terminus of the protein that render *IRC7* inactive. This variation might account for the strain variation observed in 4MMP release (Howell et al. 2005; Swiegers et al. 2009).

3MH release, on the other hand, appears to be mediated by more than one gene (Roncoroni et al. 2011; Thibon et al. 2008). It was recently demonstrated that the cystathionine β-lyase *STR3*, integrated into a commercial wine yeast under the control of a constitutive promoter, increased release of 3MH by 30 % (Holt et al. 2011). The activity of this enzyme against Cys-3MH in vitro was consistent with 3MH release being a side (non-physiological) activity, reinforcing the concept that for highly potent compounds such as 3MH, small contributions by multiple non-specific carbon–sulfur lyase enzymes may be important during winemaking.

In contrast to the growing knowledge of cysteine conjugate release, there have been no detailed studies of polyfunctional thiol release from glutathionated precursors. Based upon equivalent conversion rates, and relative abundance of precursors, it was estimated that up to 20 % of 4MMP was derived from the glutathionated precursor (Roland et al. 2010b). Glutathionylated 3MH can also be released by yeast (Grant-Preece et al. 2010; Roland et al. 2010a), but at lower efficiency than the cysteinylated precursor (Kobayashi et al. 2010; Winter et al. 2011b), the latter estimated to contribute to 3–7 % of the total 3MH found in wine (Subileau et al. 2008b). Nonetheless, this lower conversion of glutathionylated 3MH might be compensated by its high abundance, which in some juices has been reported to be up to 35 times higher than that of Cys-3MH (Capone et al. 2010).

It has been proposed that glutathionated thiol precursors enter the yeast cell via the high affinity glutathione transporter, *OPT1*, since its deletion resulted in a 2-fold decrease in the formation of 3MH in grape must (Subileau et al. 2008b). Once inside the cell, the mechanism by which the glutathionated thiol precursors are degraded has not been fully elucidated, but is likely to involve a multi-step pathway with the production of the cysteinylated form as an intermediate (Grant-Preece et al. 2010). In support of this, a known carbon–sulfur β-lyase could not directly cleave 3MH from its glutathionylated precursor (Winter et al. 2011b). Such a pathway would be analogous to catabolism of glutathione and of xenobiotic glutathione conjugates, involving sequential degradation of the tripeptide to individual amino acids (Ubiyvovk et al. 2006; Wuenschmann et al. 2010), largely in the vacuole.

Enhanced knowledge of genes involved in polyfunctional thiol precursor uptake and cleavage will provide several new targets for engineering of yeast to enhance varietal flavours. It is also important to note that, as these precursors contain amino acids, transcriptional networks involved in regulation of amino acid metabolism (nitrogen catabolite repression [NCR]) in turn affect polyfunctional thiol release. The abolition/relief of NCR by deleting the transcriptional regulator *URE2*, results in an increase in the release of both 3MH and 4MMP (Subileau et al. 2008a; Thibon et al. 2008), which was dependent on the presence of an active copy of *IRC7* and associated with an up-regulation of the *IRC7* transcript (Thibon et al. 2008).

In addition, several observational studies have highlighted natural yeast variation in capacity to release and esterify polyfunctional thiols can be harnessed to modulate wine flavour, for example see Swiegers et al. (2009). Further optimization of polyfunctional thiol release, and formation of the acetate ester of 3MH, has been achieved through co-inoculation of yeast strains and species (Anfang et al. 2009; King et al. 2008; King et al. 2010). It has also been noted that *Saccharomyces* interspecies hybrid yeast produce relatively high concentrations of polyfunctional thiols (Swiegers et al. 2009). The latter observation may prove particularly useful for development of flavour active wine yeast that produce higher concentrations of positive flavour compounds, whilst producing low levels of H_2_S (Bizaj et al. 2012).

## Future perspectives — new approaches to unravel the yeast flavour phenotype

Targeted development of yeast strains that enhance varietal wine flavours, or contribute to wine complexity, is an endeavour still in its infancy. Strain development has been mainly based on classical strain selection and modification methods, such as variant selection as a result of spontaneous mutations, mutagenesis, and hybridization (see Table 1 for examples). The advantage of these methods is that they do not give rise to products that are included in the statutory definition of genetically modified organisms (GMOs). On the other hand, these methods are not specific enough to modify wine yeast in a well-controlled manner, and they might improve some of the properties of the yeast strain, while compromising other desired traits. The use of recombinant DNA technology and genetic engineering offers the possibility to change specific properties of a yeast strain (reviewed by Carrascosa et al. 2011), but the resulting strain is a GMO. When compared with efforts to engineer other traits into *S. cerevisiae*, it is clear that only a handful of modifications have been made to yeast to specifically alter production of flavour-active metabolites (Table 1).

**Table 1:** Examples of flavour-active yeast strain development for production of saké, beer and wine

Target flavour compound(s)	Strain	Description of method	Selection/screening	Phenotype	Target gene/enzymatic activity	Reference(s)
Hydrogen sulfide (H_2_S)	Commercial wine yeast (Maurivin PDM)	Chemical mutagenesis	Screening of H_2_S production in a plate assay	Low H_2_S	MET10, MET5	(Cordente et al. 2009) (Patent PCT/AU08/01485)
	Commercial and native wine yeasts	Allele swapping (MET10-932) from naturally occurring low-H_2_S strain		Low H_2_S	MET10	(Linderholm et al. 2010) (Patent WO 2008/115759)
	Commercial wine^1^ and brewing^2^ yeast	Overexpression of O-acetyl homoserine-O-acetyl serine sulfhydrylase (MET17)		Low H_2_S	MET17	(Spiropoulos and Bisson 2000^1^; (Omura et al. 1995^2^)
	Brewing^1^ and native wine^2^ yeast	Transformation of cystathionine β-synthase (CYS4) allelic variants		Low H_2_S	CYS4	(Tezuka et al. 1992^1^; Linderholm et al. 2006^2^)
	Commercial brewer’s yeast	Inactivation of sulfite reductase		Low H_2_S, high SO_2_	MET10	(Hansen and Kielland-Brandt 1996)
	Commercial wine yeast (AWRI 1640, AWRI 1116, AWRI 1539)	Interspecific hybridization between a low-H_2_S *S. cerevisiae* strain and high-flavour *S. cerevisiae* x *S. kudriavzevii* hybrids)	Complementary selectable markers and screening of H_2_S production in a plate assay	Low H_2_S, high flavour	MET10	(Bizaj et al. 2012)
Polyfunctional thiols	Commercial wine yeast (VIN13)	Overexpression of *E. coli* tryptophanase		Increased 3MH, 4MMP	Cysteine S-conjugate β-lyase	(Swiegers et al. 2007)
	Commercial wine yeast (VIN13)	Overexpression of cystathionine β-lyase STR3		Increased 3MH	STR3	(Holt et al. 2011)
	Commercial wine yeast (Zymaflore F15)	Overexpression of β-lyase IRC7		Increased 3MH. 4MMP, 3MHA	IRC7	(Roncoroni et al. 2011)
	Commercial wine yeast (VIN13)	Overexpression of alcohol acetyltransferase ATF1		Increased 3MHA	ATF1	(Swiegers et al. 2006)
	VL3-1D (derived from commercial wine yeast VL3c)	Deletion of nitrogen catabolite repression transcriptional regulator URE2		Increased 3MH, 4MMP, 3MHA	URE2	(Thibon et al. 2008) (Patent WO 2008/068635)
Monoterpenes (de novo biosynthesis)	Haploid laboratory strain	UV mutagenesis	Resistance to nystatin and screening with radioactive mevalonate	Increased geraniol, linalool	ERG20, ERG9	(Chambon et al. 1990; Chambon et al. 1991)
	Haploid laboratory strains	Overexpression of geraniol synthase from *Ocimum basilicum*, and of farnesyl pyrophosphate synthetase ERG20 mutated allele		Increased geraniol, linalool	Monoterpene synthase, ERG20	(Fischer et al. 2011; Oswald et al. 2007)
	Wine strain T_73_-4	Overexpression of linalool synthase from *Clarkia breweri*, and deletion regulatory region of HMG-CoA reductase (HMG1)*		Increased linalool	Monoterpene synthase, HMG1	(Herrero et al. 2008; Rico et al. 2010)^*^
Higher alcohols	Diploid saké yeast (K30)	Spontaneous mutations	Resistance to leucine analog 5,5,5-trifluoro-dl-leucine	Increased isoamyl alcohol and its acetate	LEU4	(Oba et al. 2005)
	Haploid saké yeast (G1101, G1103)	Chemical mutagenesis	Resistance to leucine analog 5,5,5-trifluoro-dl-leucine	Increased isoamyl alcohol and its acetate	LEU4	(Ashida et al. 1987)
	Diploid saké yeast (Kyokai 9)	Chemical mutagenesis	Resistance to phenylalanine analogs (p- and o-fluoro-dl-phenylalanine)	Increased phenylethanol and its acetate	TYR1 (p-analog) ARO4 (o-analog)	(Fukuda et al. 1990a; Fukuda et al. 1990b)
	Haploid laboratory strain (BY4741)	Overexpression of rose phenylacetaldehyde synthase		Increased phenylethanol	Phenylpyruvate decarboxylase	(Farhi et al. 2010)
	Commercial wine strain (VIN13)	Overexpression of branched-chain amino acid transaminases BAT1 and BAT2		Increased isobutanol, isoamyl alcohol	BAT1, BAT2	(Lilly et al. 2006b)
Esters	Haploid saké yeast G1103 (derived from Kyokai 7)	Chemical mutagenesis	Resistance to fatty acid synthase (FAS) 2 inhibitor cerulenin	Increased ethyl hexonate and hexanoic acid	FAS2	(Ichikawa et al. 1991)
	Diploid saké yeast (Kyokai 7)	Allele swapping (FAS2 G1250S) from cerulenin-resistant yeast (self-cloning)		Increased ethyl hexonate and hexanoic acid	FAS2	(Aritomi et al. 2004)
	Diploid saké yeast (Kyokai 901)	Chemical mutagenesis	Screening of flavour profile of bank of mutants	Increased isoamyl acetate or ethyl hexanoate		(Arikawa et al. 2000)
	Saké yeast 2NF	Spontaneous mutations	Resistance to farnesol analog 1-farnesylpyridinium^1^ or to copper^2^	Increased isoamyl acetate	Increased alcohol acetyltransferase activity	(Hirooka et al. 2005)^1^; (Hirooka et al. 2010)^2^
	Commercial wine strains (VIN13, VIN7)^1^ Commercial lager strain (CMBS33)^2^	Overexpression alcohol acetyltransferases ATF1 and ATF2		Increased acetate esters	ATF1, ATF2	(Lilly et al. 2000)^1^; (Lilly et al. 2006a)^1^; (Verstrepen et al. 2003)^2^
	Diploid saké yeast (Kyokai 7)	Overexpression alcohol acetyltransferase ATF1(self-cloning)		Increased isoamyl acetate	ATF1	(Hirosawa et al. 2004)

This is partly due to the diversity of ‘flavour’ phenotypes and highly specialized analytical techniques required to objectively measure chemical targets linked to them. Consequently, few large-scale systematic studies have been performed to identify gene targets for modification. Indeed, screening of entire yeast deletion libraries for mutations affecting flavour compound formation has, thus far, only been applied to H_2_S production (Linderholm et al. 2008). Higher alcohol formation was probed using a targeted subset of deletion strains by Styger et al. (2011a), due to the limitations imposed by chemical analysis. Further development in the area of high throughput metabolite analyses, coupled with fermentation miniaturization (Liccioli et al. 2011) will be required before broader studies are likely to be performed. Similarly, while a proven approach to map the genetic variation corresponding to phenotypic variation in *S. cerevisiae*, including wine yeast phenotypes (Ambroset et al. 2011), quantitative trait loci (QTL) mapping has seen limited application in understanding complex ‘flavour phenotypes’ involving multiple flavour compounds. QTL guided breeding has been successfully applied to minimize acetic acid and H_2_S production, and the release of volatile phenols from odourless precursors (Marullo et al. 2006, 2007), highlighting the potential of this approach.

With the development of low-cost, high-throughput DNA sequencing technologies, the genomes of several wine yeasts have become available (Borneman et al. 2008, 2011, 2012; Novo et al. 2009). Comparative genomic studies of yeast strains have already shown that not only there is a substantial nucleotide variation within the *S. cerevisiae* species, but also the presence of several regions of DNA that are specific and are predicted to encode proteins that are unique to certain industrial strains. An example are the presence of novel aryl-alcohol dehydrogenase (AAD) proteins in the wine strain AWRI796, when compared with other wine strains, which may have a direct impact on the production of higher alcohols and other flavour compounds during fermentation (Borneman et al. 2011). It is likely, though, that ‘flavour phenotype’ variation amongst wine yeast strains will be determined by differences in transcriptional network regulation. It has been proposed that some of the primary evolutionary targets of strain diversification are transcription factors and their binding sites (Dermitzakis and Clark 2002). Data show that although *S. cerevisiae* and *Saccharomyces mikatae* have similar genome sequences, they are significantly different in their transcription factor binding profiles (Borneman et al. 2007a, b). Recent studies have provided some insight into transcriptional networks involved in flavour compound formation (Rossouw et al. 2008, 2009) by wine yeast, and it was shown that the metabolic phenotype of a strain can be shifted by changing expression levels of individual (key) transcription factors (Rossouw et al. 2012).

Increased availability of genome sequences in combination with QTL studies will also reveal allelic variants of genes known to be involved in flavour compound formation, that may explain variation in ‘flavour phenotypes’ amongst wine yeast strains. Hydrogen sulfide formation by wine yeast was recently linked to allelic variants of *MET5* and *MET10* (Cordente et al. 2009; Linderholm et al. 2010), while formation of the polyfunctional thiol 4MMP relies upon an apparently rare allele of *IRC7* (Roncoroni et al. 2011). Investigation of the impact known allelic flavour gene variants (Linderholm et al. 2006; Linderholm et al. 2010) have upon ‘flavour phenotypes’, while extending the search for flavour-active alleles beyond *S. cerevisiae*, has the potential to greatly expand the toolkit of synthetic biologists and provide options for multi-yeast starter cultures. Recent examples where flavour-impact of non-*S. cerevisiae* wine yeast have been evaluated include *S. bayanus* (Masneuf-Pomarede et al. 2010), *Torulaspora delbruekii* (Renault et al. 2009) and *Pichia kluyverii* (Anfang et al. 2009). As the genomes of non-*Saccharomyces* wine yeasts are sequenced and made available, the understanding of the global wine metabolic network will provide wine yeast strain developers with a broader range of options to confer desirable ‘flavour phenotypes’.

Indeed, looking over the horizon at emerging technologies and how they might impact future strain development strategies, it may soon be possible to bring *all* ‘flavour-active’ genes, or interesting alleles from diverse species, together in a single ‘re-programmed’ yeast strain. Recently, a chemically synthesized chromosome of the bacterium *Mycoplasma mycoides* — all 1.08 mega basepairs of its DNA — was successfully transplanted into a closely related bacterial cell, *Mycoplasma capricolum* (Gibson et al. 2010)*.* This marked a world-first: a ‘synthetic’ genome, created in silico, giving life to another living organism with no ancestor. The emerging field of synthetic biology is revolutionizing biotechnology, providing the means to systematically reprogram the genetic makeup of biological systems using ‘off-the-shelf’ functional genetic modules. Within such a context, wine yeast ‘flavour phenotypes’ could be effectively uncoupled, making it possible to develop yeast strains that produce wines with flavour profiles that are difficult to achieve currently — or indeed provide the means to rapidly develop new wine styles. Pending societal acceptance of wines made using GM organisms, the potential for future advances will be limited only by knowledge of flavour compounds and their formation.
